# When fixation creates fiction

**DOI:** 10.7554/eLife.85671

**Published:** 2023-02-16

**Authors:** Judith Miné-Hattab

**Affiliations:** 1 Laboratory of Computational and Quantitative Biology, CNRS, Institut de Biologie Paris-Seine, Sorbonne Université Paris France

**Keywords:** liquid-liquid phase separation, fixation, live-cell single-molecule imaging, intrinsically disordered proteins, condensates, cross-linking, None

## Abstract

A chemical regularly used to image cells can dramatically alter the way cellular compartments called condensates look under the microscope.

**Related research article** Irgen-Gioro S, Yoshida S, Walling V, Chong S. 2022. Fixation can change the appearance of phase separation in living cells. *eLife* **11**:e79903. doi: 10.7554/eLife.79903.

The nucleus of mammalian cells is crowded with millions of proteins, about 30% of which are organized into sub-compartments called condensates. Inside these membrane-less structures, specific proteins are highly concentrated while others are excluded, creating a micro-environment that favors or impedes particular biological tasks (Miné-Hattab and Taddei, 2019).

How these condensates are formed, maintained and disassembled is an active field of research in cell biology. In recent years, it has been proposed that some condensates emerge through a biochemical process known as liquid-liquid phase separation (Hyman et al., 2014). In this model, nucleic acids, chromatin and certain proteins come together because they feature specific domains which can form weak chemical bonds. Condensates generated through this process are highly dynamic: they move and fuse in the cell, with proteins freely diffusing within the compartments, as well as transitioning in and out of them (Altmeyer et al., 2015; Miné-Hattab et al., 2021; Miné-Hattab et al., 2022). Importantly, the liquid nature of some condensates seems critical for them to work properly, as this feature is sometimes altered in cells from diseased tissues (Wang et al., 2021).

Scientists commonly study liquid-liquid phase separation by tracking proteins that have been tagged with a fluorescent marker. However, doing this in living cells is sometimes technically challenging, especially if scientists want to work at endogenous proteins concentration to preseve expression levels. Instead, researchers often ‘fix’ the cells before imaging them by applying chemical treatments which hold their molecules in place. This allows researchers to take a snapshot of the cells in vivo at specific points in time.

However, whether fixation preserves the distribution of molecules and the appearance of cells remains poorly understood. For example, it is possible that this method fails to capture molecular events which take place faster than the several minutes it takes fixative agents to move through and immobilize molecules in the cell. In addition, proteins vary in how quickly they stop moving once they are exposed to the fixative molecules. Fixation may therefore not provide an instantaneous snapshot of a cell. In particular, it may be ill-suited to capture highly dynamic structures such as condensates. Now, in eLife, Shasha Chong and colleagues at the California Institute of Technology – including Victoria Walling and joint first authors Shawn Irgen-Gioro and Shawn Yoshida – report results which show that this method may not be preserving the appearance of liquid-liquid phase separation in human cells (Irgen-Gioro et al., 2022).

The team created genetically altered cells which over-expressed specific protein regions involved in liquid-liquid phase separation, which are known as intrinsically disordered regions. The distribution of fluorescently tagged proteins known to form liquid-liquid phase separation was then imaged before and after cells had been fixed with paraformaldehyde, a commonly used compound which cross-links neighboring molecules in a non-selective way.

The experiments showed that paraformaldehyde could strongly alter the appearance of liquid-liquid phase separation, but that its effect differed depending on the protein being observed. It could sometimes have no significant impact, but it could also increase or decrease the size of existing puncta (the microscopic structures detected during imaging which are interpreted as being condensates). In extreme cases, puncta present in living cells could even appear or disappear entirely after fixation. These changes remained even after the addition of glutaraldehyde, a compound known to reduce fixation artefacts in the cell membrane.

What could explain this puzzling variety of fixation artefacts? Irgen-Gioro et al. observed that the effects of paraformaldehyde could be reversed when cells had first been exposed to glycine, a molecule which alters fixation rates. This led them to propose a model in which the type of artefacts created by fixation relied on the balance between protein interactions and fixation dynamics in a liquid-liquid phase separation system.

Three factors which controlled the fixation output were identified: the rate at which proteins were exchanged between the condensate and the rest of the cell; how fast molecules were fixed inside the condensate; and how fast they were fixed outside of it. When the exchange rate is higher than fixation rates, changes in the size and number of puncta depend on whether molecules outside or inside these structures are fixed first (Figure 1). If proteins inside the condensate are free to move but those in the cell are already fixed by the paraformaldehyde, the compartments appear to shrink or even disappear if proteins in the cell are ‘frozen’ first since, in this context, molecules can still leave the compartment, but not enter it. Conversely, puncta grow and multiply if their internal proteins are fixed in place when the ones outside remain able to move in. Critically, Irgen-Gioro et al. identified that paraformaldehyde preserves proteins organization when internal and external fixation rates are much faster than the rate at which proteins interact and are exchanged in and out of condensates. In other words, the structures are preserved if molecules are fixed ‘instantaneously’ before they have a chance to leave or enter the compartment.

**Figure 1.**
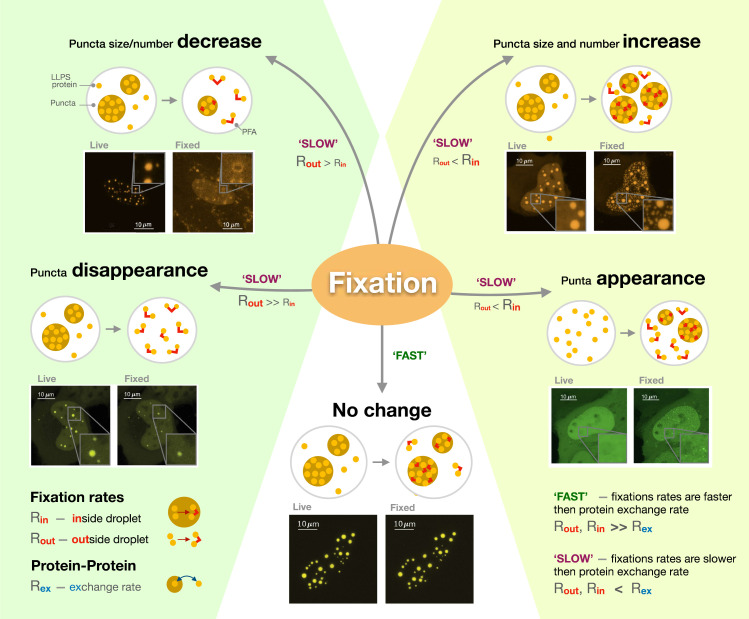
The different artefacts of paraformaldehyde fixation. Irgen-Gioro et al. imaged cells carrying proteins (labeled LLPS proteins) genetically manipulated to be more likely to form condensates (known as puncta; brown circles) through liquid-liquid phase separation, before (live) and after (fixed) having been exposed to paraformaldehyde (PFA; red lines). They then established a model which captures the different types of artefacts the fixative agent can create. The model showed that PFA can cause the size and number of puncta to decrease (top left), increase (top right) or remain unchanged (bottom). In extreme cases, fixation can lead to the disappearance of puncta (bottom left) or the creation of new puncta (bottom right). These artefacts are formed when the rate at which proteins are exchanged in and out of puncta (R_ex_) is slower than fixation rates (SLOW); whether fixation happens more rapidly inside (R_in_) or outside (R_out_) the condensate then determines which type of artefacts will occur. However, protein location will be preserved if fixation is much faster than molecular exchange (FAST).

Image credit: Olga Markova.

This work reveals that fixation is an active player in intracellular dynamics, interacting with proteins in the cell and influencing their organization. The cell biology community should be aware of this study when they interpret their results. In the future, other fixative agents should also be tested besides paraformaldehyde, and new compounds should be developed that can minimize fixation artefacts.

## References

[bib1] Altmeyer M, Neelsen KJ, Teloni F, Pozdnyakova I, Pellegrino S, Grøfte M, Rask MBD, Streicher W, Jungmichel S, Nielsen ML, Lukas J (2015). Liquid demixing of intrinsically disordered proteins is seeded by poly(ADP-ribose). Nature Communications.

[bib2] Hyman AA, Weber CA, Jülicher F (2014). Liquid-liquid phase separation in biology. Annual Review of Cell and Developmental Biology.

[bib3] Irgen-Gioro S, Yoshida S, Walling V, Chong S (2022). Fixation can change the appearance of phase separation in living cells. eLife.

[bib4] Miné-Hattab J, Taddei A (2019). Physical principles and functional consequences of nuclear compartmentalization in budding yeast. Current Opinion in Cell Biology.

[bib5] Miné-Hattab J, Heltberg M, Villemeur M, Guedj C, Mora T, Walczak AM, Dahan M, Taddei A (2021). Single molecule microscopy reveals key physical features of repair foci in living cells. eLife.

[bib6] Miné-Hattab J, Liu S, Taddei A (2022). Repair foci as liquid phase separation: evidence and limitations. Genes.

[bib7] Wang B, Zhang L, Dai T, Qin Z, Lu H, Zhang L, Zhou F (2021). Liquid-liquid phase separation in human health and diseases. Signal Transduction and Targeted Therapy.

